# Setting Sails for Your Harbor: Navigating Beyond NEET Status Through Self‐Efficacy and Career Decidedness

**DOI:** 10.1002/jad.70051

**Published:** 2025-09-16

**Authors:** Gloria Willhardt, Ute‐Christine Klehe, Miriam Schäfer

**Affiliations:** ^1^ Department of Work and Organizational Psychology Justus‐Liebig‐University Giessen Germany; ^2^ Department of Human Resource Development Justus‐Liebig‐University Giessen Germany

**Keywords:** career decidedness, NEET, school‐to‐work‐transition, self‐efficacy, unemployment, youth

## Abstract

**Introduction:**

A successful transition from school to further employment, education, or training is central to avoiding early unemployment with its dire consequences for young people and society. Yet, some youths struggle with this transition and fall into a NEET‐status, “not in employment, education, or training.” By studying the temporal dynamics of such NEETs' career related self‐efficacy and career decidedness across four waves, the current study aims to gain an understanding of how such young people may transition back out of NEET status.

**Methods:**

For this purpose, the current study followed *N* = 264 NEETs in Germany (aged 15–25; 42.6% female, mean age 18.41 years) up to four measurement points between 2014 and 2016 in a cross‐lagged panel design to trace their career‐related self‐efficacy and career decidedness as predictors of their eventual ability to leave the NEET status.

**Results:**

Results indicate that while self‐efficacy and career decidedness covaried when studied crossectionally, contrary to conceptual predictions, neither self‐efficacy nor career decidedness impacted each other across time. Further, the more decided NEETs felt towards the end of the study's period, the higher their chance of exiting the NEET status by finding employment, returning to school for a degree, or starting an apprenticeship or training.

**Conclusion:**

This study sheds light on the interplay between self‐efficacy and career decidedness over multiple measurement points and how youths with otherwise bleak outlooks decide on their future career and possibly enter more favorable career trajectories. Practical implications include advice for programs targeting younger people.

If one does not know to which port one is sailing, no wind is favorable

(Seneca ca. 65 AD).

Transitioning from school to work is a pivotal step in young people's vocational development (Dietrich et al. 2012). Successful school‐to‐work transitions (STWT) foster labor market integration, supportive psychosocial networks, and long‐term career prospects (Akkermans et al. 2021). However, some youths struggle with this transition (e.g., Burgess et al. 2003), prompting research attention from vocational, educational, and social psychology.

One key theme across these research streams is the importance of young people reaching career decidedness—the certainty one has about one's occupational course (Jaffe 1998) and an indicator of career intentions. Reasoning theories^1^ like the theory of reasoned action (TRA; Ajzen and Fishbein 1980) and the theory of planned behavior (TPB; Ajzen 1991) highlight the importance of intentions in predicting behavior, and career decidedness in particular provides young people with direction for their careers.

A second key theme is career‐related self‐efficacy—the conviction that one can perform the actions needed to pursue one's career (Lent and Hackett 1987). Indeed, self‐efficacy is generally understood as an important predictor for career decidedness (Betz and Voyten 1997; Taylor and Betz 1983) and for securing an internship, a job, or any occupation at all (Hong et al. 2021). This assumption again aligns well with reasoning theories like the theory of planned behavior (Ajzen 1991), which views self‐efficacy as a primary component of people's perceived behavioral control, a core predictor of intentions and subsequent action, yet also with diverse careers theories: For example, Gottfredson (2005) argues that young people's career choices depend on perceived risks and effort needed, implying that young people shy away from options they lack self‐efficacy in. Career construction theory (CCT; Savickas 2005, 2013) and social cognitive career theory (SCCT; Lent and Brown 2013) both view self‐efficacy as a key resource fostering decidedness and related outcomes.

Yet, most studies linking self‐efficacy to career decidedness are cross‐sectional (e.g., Taylor and Betz 1983). In contrast, longitudinal findings are mixed (Lent et al. 2019) or null (Creed et al. 2006), questioning a core assumption of the above theories on the link between self‐efficacy and career decidedness, that is, the long‐assumed causal pathways. Based on this mismatch between conceptual frameworks and empirical findings, we were interested in the stronghold of the positive link between career‐related self‐efficacy and career decidedness across time.

The current study systematically addresses this question among a group of young people with a pressing need to decide on their future career path. “*NEETs*” (not in employment, education, or training) are young people, usually aged 15 to 24, who exit school (with or without a degree) and fail to enter further education (e.g., college, apprenticeships) or the labor market, thus lacking a clear career path (see e.g., Maguire 2015). A diverse yet specific sub‐group of the unemployed (Carcillo et al. 2015), NEETs are at high risk for a downward spiral of unemployment (Carcillo et al. 2015), reduced earnings (Mroz and Savage 2006), and diminished job satisfaction, happiness, and health (Scarpetta et al. 2010).

We follow NEETs in Germany across a 10‐month interim period. Exploring the time‐lagged relationship between NEETs' self‐efficacy and career decidedness, we expect self‐efficacy and career decidedness to influence each other across time and to improve NEETs' future occupational status by helping them exit their NEET status (see Figure 1 for the development of the full study model).

**Figure 1:**
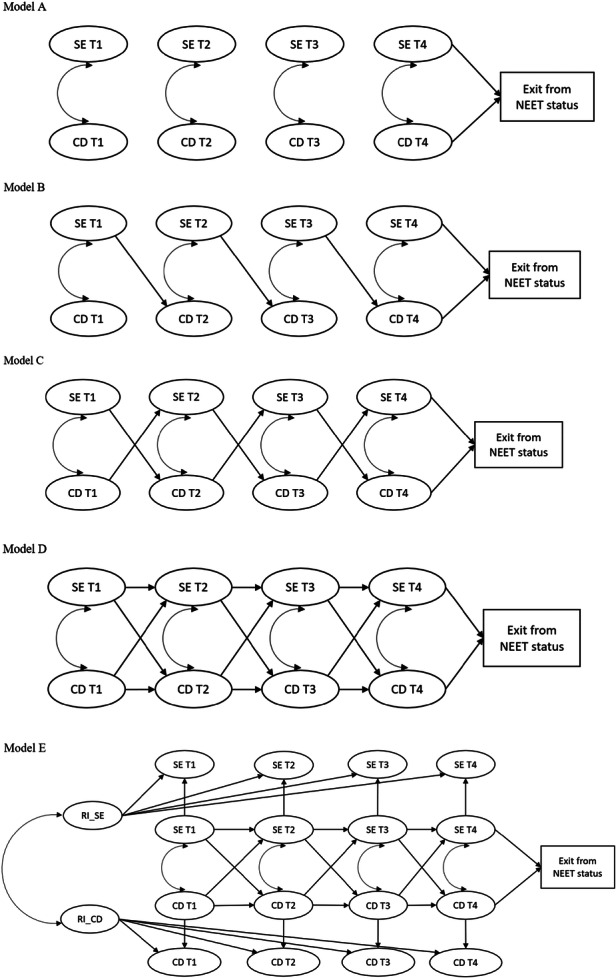
Overview of proposed study models. CD = Career Decidedness, SE = Self‐Efficacy. Model A: Correlations only design. Model B: Predictive design. Model C: Predictive design with reciprocal paths. Model D: Cross‐Lagged Panel Model (CLPM) design with autoregressive paths. Model E: RI_CD = Random Intercept for Career Decidedness, RI_SE = Random Intercept for Self‐Efficacy. Random Intercepts denote the between‐person part of the model.

Together, this paper makes two theoretical and practical contributions. First, it contributes to the literature on self‐efficacy and career decidedness (Betz and Voyten 1997; Creed and Patton 2003; Creed et al. 2006; Taylor and Betz 1983; Sheu 2023; Lent et al. 2019) by addressing the stability and dynamic relationship between these two variables over a 10‐month period with multiple measurement points, using increasingly complex study designs that pay credit to alternative possible explanations that may otherwise explain for the positive relationship found in previous—mostly cross‐sectional—research. With this, we hope to identify the true nature of this relationship, to shed light on the current mismatch in empirical findings, and to contribute to existing conceptual frameworks (Gottfredson 2005; Savickas 2005; Lent and Brown 2013; Lent et al. 1994; Ajzen 1991), while addressing the relative scarcity of longitudinal research on this relationship (Creed et al. 2006; Lent et al. 2019). Observing changes over time allows for stronger inferences regarding temporal directionality and within‐person changes, which cross‐sectional designs cannot capture.

In this context, we further study NEETs in particular, as they are arguably in a particularly dire situation. Typically, NEETs represent a severely challenged and easily stigmatized subgroup with a bleak future outlook and few job prospects. Given their unstable labor market position and disrupted STWTs, their career decision‐making is particularly relevant and urgent to prevent a lifetime of un‐ and underemployment. With this, we second, also contribute to the literature on NEETs, a highly relevant yet greatly understudied group of youth, by examining whether previously found beneficial effects of career decidedness and self‐efficacy among graduates (Cheunga et al. 2019; Ye 2014) also hold true for this less fortunate group. Much research has been devoted to young people in preparation or undergoing regular career transitions in school (Creed et al. 2006), college, or upon graduation (Uthayakumar et al. 2010). Yet, we need far more research on vulnerable youths who might have different needs than the average college graduate. With this study, we aim to explore mechanisms to help NEETs break out of the (self‐reinforcing) cycle of intergenerational poverty.

## 1 Theoretical Background

### 1.1 NEETs: Youth Unemployment

Arriving at a viable career decision and entering employment, education, or training is a critical developmental step. Yet, about one in eight young people in the EU and one in five globally (International Labour Office 2020) struggle with this transition, becoming NEET—not in employment, education, or training (Mascherini 2018). While temporary unemployment is common among youth (International Labour Office 2020), prolonged disconnection from school or work increases the risk of long‐term labor market exclusion (Eurofound et al. 2012).

The hardships and costs associated with unemployment affect all, NEETs, their families, and society (Eurofound et al. 2012) through lost early built human capital, lower lifetime earnings, less access to decent work (Chandola and Zhang 2018; Hillmert 2006; Searle 2021), and discouragement from labor market participation (Eurofound et al. 2012). NEET status thus fosters later un‐ and underemployment, which also impairs the career prospects of one's own children, as NEETs are more likely to come from disadvantaged backgrounds (Mascherini 2018; Berloffa et al. 2018), perpetuating intergenerational disadvantage. Supporting NEETs through this vulnerable phase is thus essential—both for their individual career development and society overall.

### 1.2 Self‐Efficacy and Career Decidedness

The school‐to‐work transition is a prolonged and dynamic process embedded in broader career development (Lent et al. 1999). One of the first tasks, typically in late adolescence, shortly before or after leaving the formal general educational system (see Gordon 1998), is deciding which career to pursue (Marciniak et al. 2022; Arnold 1989; Super 1981). Career decidedness refers to having and being certain about one's career goal (Greenhaus et al. 1995) and can have substantial benefits for psychological well‐being and other relevant outcomes (Arnold 1989; Uthayakumar et al. 2010) and ultimately help to master the STWT.

When predicting career decidedness, self‐efficacy, one's belief in one's ability to effectively complete a particular task (Bandura 1977), seems of particular importance. Theories from different conceptual backgrounds emphasize the role of self‐efficacy in deciding upon a vocational course of action and in persisting and investing effort into this pursuit (Lent et al. 1994; Lent et al. 1999; Deci and Ryan 1985). Reasoning approaches, like the theory of planned behavior (Ajzen 1991), generally emphasize the importance of perceived behavioral control—how strongly a person believes they can carry out a particular behavior—for shaping intentions and actions. Among career theories in particular, Gottfredson's developmental theory of *circumscription and compromise* proposes that while teenagers understand the social hierarchy of professional positions, they “rule out occupations that are too difficult for them to enter with reasonable effort or that pose too high a risk of failure if they try” [tolerable‐effort boundary] (Gottfredson 2005, 79). Also *career construction theory* (CCT) proposes that career adaptability, an “individual's readiness and resources for coping with current and anticipated tasks of vocational development” (Savickas 2005, 46) fosters adapting responses, such as career‐related self‐efficacy, which then foster adaptation results, such as career decidedness (Savickas 2005, 2013; cf. Hirschi et al. 2015). Finally, *social cognitive career theory* (SCCT; Lent and Brown 2013) proposes that career‐related self‐efficacy facilitates young peoples' career decidedness, which then is needed for positive career outcomes, such as getting (back) into employment, or getting a degree.

Indeed, past research with college students found significant negative relationships between students' overall career decision‐making self‐efficacy and their level of career indecision. Penn and Lent (2019) further showed how self‐efficacy mediated the relationship between certain personality variables and career decidedness.

Yet, while evidence for the interplay between self‐efficacy and career decidedness seems plenty, the respective studies are mostly cross‐sectional (Betz and Voyten 1997; Taylor and Betz 1983; Lent et al. 2016). When addressing this relationship with more advanced methodological approaches, results are far less convincing^2^: Using a two‐wave design, Creed et al. (2006) studied the career decision‐making self‐efficacy and career indecision of schoolchildren at around age 14 (grade 8) and again at age 16 (grade 10). While they found stable autoregressive paths, meaning that both self‐efficacy and career decidedness predicted themselves 2 years into the future, they found no cross‐lagged relationships, which speaks against the common assumption that self‐efficacy fosters career decidedness or vice versa—at least when simultaneously controlling for their respective starting values. That said, 2 years is indeed a long time‐period, particularly during students' turbulent puberty years with their numerous developmental experiences. Lent et al. (2019) studied the longitudinal relationship between self‐efficacy and career decidedness across three time points spanning roughly 7 months, this time among college students. Accounting for the autoregressive stability of these two variables, they indeed found support for self‐efficacy predicting decidedness from time two to time three but not from time one to time two. Further, they found support for decidedness predicting self‐efficacy from time one to time two, yet did not test this relationship for time two to time three. Finally, Sheu (2023) used an even shorter timelag and showed that college students' career decision self‐efficacy, aggregated across five time points over the span of 10 weeks, related positively to their career decidedness gathered only at time five. They concluded that individual differences in career decision self‐efficacy can influence career decidedness, but did not control for students' starting career decidedness and didn't study how those variables might influence one another across time.

Thus, we aim to re‐examine these surprisingly mixed effects in the longitudinal relationship between career‐related self‐efficacy and career decidedness, yet with a shorter time‐lag and among a type of youth population to whom self‐efficacy and decidedness may be of critical importance. Given their scant educational and labor market prospects paired with a pressing need to arrive at a career decision in the near future, NEETs may easily suffer from a lack of both self‐efficacy and decidedness, and deciding about their occupational future and finding employment or further training or education is of utmost importance.

Based on theoretical thinking above, we expect a positive relationship between NEETs' self‐efficacy and their career decidedness (H1a; Figure 1A). More so, the logic of both reasoning theories like the TPB (Ajzen 1991) and career related theories such as Gottfredson (2005) theory of circumscription and compromise, the CCT (Savickas 2005), and SCCT (Lent and Brown 2013) suggest a temporal prediction within this relationship in that career related self‐efficacy should foster subsequent career decidedness (H1b; Figure 1B). Further, following the assumptions previously made by Creed et al. (2006) and the theoretical logic of SCCT (Lent and Brown 2013), we also expect a feedback loop from career decidedness to participants' later career‐related self‐efficacy in return. Career decidedness, after all, will foster actions and experiences that will then serve as learning experiences for future self‐efficacy (H1c; Figure 1C). Yet, as Creed et al. (2006) and Lent et al. (2019) already noted, such relationships should not only emerge in prospective designs testing for the effect of self‐efficacy at one time point on young people's career decidedness at a later point and vice versa. To draw any causal conclusions, these relationships should also hold when controlling for participants' starting values of self‐efficacy and career decidedness, respectively, thus controlling for autoregressive stability (H1d; Figure 1D). Finally, common depictions of theories relating to both self‐efficacy and decidedness based on classic cross‐lagged models do not differentiate within‐person variance from between‐person variances, even though such differentiation is relevant for making causal attributions across time (Hamaker et al. 2015; Lent and Brown 2013). That means one needs to differentiate the variance attributed to participants' self‐efficacy overall and career decidedness overall from the temporal changes in these constructs from one time to the next if one wants to truly test whether it is self‐efficacy at one particular point in time that predicts career decidedness at a later point in time (and vice versa; H1e; Figure 1E).

Taken together, we thus propose:

Hypothesis 1a There is a positive cross‐sectional correlational relationship between NEETs' career‐related self‐efficacy and career decidedness.

Hypothesis 1b There is also a positive, temporally predictive relationship from NEETs' career‐related self‐efficacy to subsequent career decidedness.

Hypothesis 1c There is a positive, temporally predictive relationship from NEETs' career‐related career decidedness to subsequent self‐efficacy.

Hypothesis 1d The positive, cross‐lagged relationships (H1b, H1c) also occur when controlling for autoregressive stability for self‐efficacy and career decidedness.

Hypothesis 1e The positive, cross‐lagged relationships (H1b, H1c) also hold when additionally controlling for between‐person variance.

### 1.3 NEETs' Future Career Prospective

The primary goal of any program for NEETs is to help them exit NEET status by entering employment, education, or training. Accordingly, all three outcomes—taking up a job and earning an income (employment), pursuing further academic credentials (education), or entering school‐based training for vocational qualifications (training)—are desirable.

We assume that both self‐efficacy and career decidedness will help NEETs succeed in exiting their NEET status. According to SCCT (Lent and Brown 2013), for example, self‐efficacy aids the pursuit of career‐related behaviors. Indeed, previous research found job search self‐efficacy to negatively predict the number of interviews people had before finding employment, suggesting that people with higher self‐efficacy are more confident and thus more efficient in their search (Moynihan et al. 2003). Also career decidedness—reflecting a formed and stable intention—may guide action and persistence in pursuing opportunities (Ajzen and Fishbein 1980; Ajzen 1991), thereby shaping more favorable career trajectories. Together, career decision self‐efficacy and goals have shown to predict subsequent career planning over a 6‐month period (Rogers and Creed 2011), and higher career decidedness in a group of recent graduates has been linked to less resignation from a job (Earl et al. 2011). This leads us to our final hypothesis:

Hypothesis 2 NEETs final (a) self‐efficacy and (b) career decidedness positively relate to their future career prospectives.

### 1.4 Methods

#### 1.4.1 Participants and Procedure

In the German vocational training system, most formal education or training programs start between August and October. Youths who miss this window often face a year‐long wait before the next opportunity, with few meaningful interim options^3^. As these NEETs are by definition not in employment, education, or training, they cannot be reached through the usual channels of schools, universities, or the workplace. We therefore turned to an adult education provider that offers interim courses for this target group. Participants were recruited in two cohorts (2014–2016) at three program locations in central Germany. The provider's main objective is to keep young people engaged, maintaining a structured routine, raising training levels, and fostering job‐related behaviors. Consequently, participation is usually mandatory for those receiving social benefits, serving as a low‐threshold intervention aimed at social inclusion rather than academic advancement.

Our sample included 264 participants (151 male) aged 15–25 years (M = 18.4). Educational backgrounds varied: three participants had special needs education, 28 had no formal schooling, 119 had completed basic secondary education (typically 9 years), 110 had intermediate secondary education (usually 10 years), and four held vocational baccalaureate diplomas.

Participants were recruited via program officials. Participation was entirely voluntary and based on informed consent. At course start, all youths received standardized information letters detailing the study's purpose, questionnaire procedure, and incentives. Those who agreed to participate signed a consent form; minors also required written parental permission. They could opt‐out at any time without consequences. Participants received complimentary sweet snacks while responding to the surveys and a €10 voucher for completing the study. Those who did not consent to participate in this study simply did not receive any questionnaires and were thus not partaking. To ensure confidentiality, each youth was assigned a pseudonymous code based on location, initials, birth month, and birthplace prefix, allowing longitudinal data linkage while maintaining anonymity.

Each cohort participated for up to 10 months. Shorter participation periods were common and expected, given the flexibility for individuals to join the program at any time. On average, NEETs remained in the program for about 7 months. Early departure could reflect both positive (e.g., employment, education) or negative outcomes (e.g., expulsion, dropout). Data were gathered on site at four measurement points set 2.5 months apart via paper‐and‐pencil questionnaires administered by a trained university research assistant. Sealed responses were taken to the university to later be transcribed to SPSS. At T2 we reached *N* = 197, at T3 *N* = 145, and at T4 *N* = 101 responses. Dropout analyses indicated no effects of age and gender. Participants were more likely to drop out before T2 when they were less educated and more decided about their future careers, whereas drop‐out between T3 and T4 was higher for NEETs with higher self‐efficacy.

#### 1.4.2 Measures

We assessed NEETs self‐efficacy and career decidedness at four time points. This assessment took place along with several other questionnaires with importance to the program but not to this study. All questionnaires were applied in German.

#### 1.4.3 Self‐Efficacy

We used Hirschi's (2009) eight‐item scale, specifically developed to measure young peoples' career choice self‐efficacy and self‐efficacy related to the search for an apprenticeship. The scale proved particularly advantageous as it had already been developed in German, eliminating the need for backtranslation. Additionally, Hirschi's validation of the scale had been conducted using a sample of German‐speaking youths.

NEETs indicated on a 5‐point‐Likert scale how confident they felt in displaying career‐related behaviors such as making satisfactory career choices or effectively mastering a job interview. A sample item is “I know exactly what I need to do to make a successful career decision”. Cronbachs' alpha ranged from 0.858 to 0.885.

#### 1.4.4 Career Decidedness

We used the six‐item Career Decidedness Scale (Lounsbury et al. 2005), asking for participants' level of agreement on a 5‐point‐likert scale to items like “I am sure about what I eventually want to do for a living.” We used Brislin's (1970) back‐translation method to be able to apply the scale in German. The Career Decidedness Scale represents a valid measure for assessing career decidedness in young people, with good convergent validity (Lounsbury et al. 1999; Lounsbury et al. 2005). Further construct validation has been provided by Lounsbury and Gibson 2002, who elaborated on the scale's robustness within work‐based personality measurement systems. Moreover, Lounsbury et al. (1999) reported excellent internal consistency with a Cronbach's alpha of 0.95. These prior validations support the reliability and concurrent validity of the scale, justifying its use in the current study. Cronbachs' alpha in our study ranged from 0.778 to 0.841.

#### 1.4.5 NEETs' Future Prospects

After the 10‐month study period, program officials provided feedback about each NEETs' whereabouts. Based on this information, we coded whether participants remained NEET (coded as 0; e.g., discontinuation of the program, termination due to breach of contract, further unemployment, and no clear plan for their future) or had exited NEET status (coded as 1; i.e., secured an apprenticeship, a signed job contract, or the decision to go back to school). The mean of this binary variable was *M* = 0.50.

### 1.5 Data Analysis

All models were estimated using MPlus 8.2 (Muthén and Muthén 2017). We ran a series of nested models of growing complexity which represented each of the assumptions outlined in hypothesis 1a to 2b. Self‐efficacy and career decidedness at each time point were modeled as latent variables measured by three parcels each for each measurement point using the item‐to‐construct balance strategy (Little et al. 2002) to create the measurement model. Parceling helped reduce model complexity while retaining measurement validity—especially important for later models with higher parameter counts. Latent variables were defined with one factor loading fixed to 1 to adequately depict the within‐person variability in all models (for further information see Hamaker 2018). As all models were nested, we systematically added the essential paths to progress to the next step while keeping all other paths. Model A (see corresponding Figure 1A) tested Hypothesis 1a and only consisted of the four latent variables of self‐efficacy and career decidedness, each, correlating with each other at each time point, and self‐efficacy and career decidedness at time four relating to NEETs' future prospects, that is, the exit from NEET status. For Model B, testing Hypothesis 1b, we added the predictive paths from self‐efficacy at one time‐point to career decidedness at the subsequent time‐point. For Model C, testing Hypothesis 1c, the respective paths from career decidedness to subsequent self‐efficacy were added. In Model D, testing Hypothesis 1 d, we additionally addressed autoregressive stability by incorporating the prediction of each variable by its own preceding value, thereby arriving at a regular Cross‐Lagged Panel Model (CLPM). This approach mirrors that of Creed et al. (2006) in their two‐wave study. In our final Model E, testing Hypothesis 1e, we analyzed a Random‐Intercept Cross‐Lagged Panel model (RI‐CLPM) for multiple indicators (Mulder and Hamaker 2021), to estimate the effect of NEETs between person variance of self‐efficacy and career decidedness. In contrast to the common CLPM, the RI‐CLPM is able to account for stability effects in the measured variables and, therefore, better separates within‐person‐ from between‐person differences (Hamaker et al. 2015; Mund and Nestler 2019). Since we assumed that both self‐efficacy and career decidedness are comprised of both stable trait‐like (between‐person) parts and malleable (within‐person) parts, it made sense to analyze those parts separately from one another. Thus, to model the stable compounds of self‐efficacy and career decidedness for Model E, we added two latent random intercepts, one for each construct (see Supplement A for a more elaborate explanation of the computation of the RI‐CLPM).

## 2 Results

### 2.1 Preliminary Analyses

A confirmatory factor analysis in Mplus (Muthén and Muthén 2017) supported the proposed measurement model with eight latent factors—one each for self‐efficacy and career decidedness at each of the four measurement points. With average factor loadings ranging from 0.70 (career decidedness T4) to 0.91 (self‐efficacy T1), the model showed a good fit (CFI = 0.955, TLI = 0.947, RMSEA = 0.044, SRMR = 0.063).

Table 1 shows the descriptive statistics and intercorrelations of the study variables. Self‐efficacy and career decidedness showed stable, positive relationships with each other within every wave; longitudinally, however, only career decidedness consistently related to subsequent self‐efficacy, while self‐efficacy linked to later decidedness only from the first wave. Career decidedness, furthermore, positively related to NEETs exiting the NEET status, with the strength of this relationship increasing across measurement points.

**Table 1:** Descriptive statistics and correlations for study variables.

Variable	N	M	SD	1	2	3	4	5	6	7	8	9	10	11
1. Sex^a^	264	0.57	0.50	—										
2. Age (Years)	264	18.39	1.89	−0.01	—									
3. SE t1	259	3.62	0.75	−0.06	0.05	(0.88)								
4. SE t2	198	3.63	0.76	−0.09	0.08	0.65^d^	(0.87)							
5. SE t3	145	3.75	0.68	0.00	−0.01	0.48^d^	0.61^d^	(0.86)						
6. SE t4	101	3.74	0.74	−0.10	−0.03	0.35^d^	0.36^d^	0.60^d^	(0.86)					
7. CD t1	259	3.25	0.90	−0.04	−0.01	0.48^d^	0.24^d^	0.20^c^	0.02	(0.84)				
8. CD t2	198	3.35	0.95	−0.16^c^	0.04	0.36^d^	0.50^d^	0.19^c^	0.11	0.50^d^	(0.83)			
9. CD t3	145	3.62	0.83	−0.04	0.06	0.19^c^	0.15	0.50^d^	0.36^d^	0.36^d^	0.28^d^	(0.83)		
10. CD t4	101	3.79	0.81	−0.24^c^	0.01	0.21^c^	0.06	0.18	0.37^d^	0.23^c^	0.19	0.52^d^	(0.78)	
11. NEETs exit^b^	245	0.50	0.50	−0.10	−0.01	0.06	0.12	0.05	0.19	0.09	0.19^d^	0.20^c^	0.43^d^	—

*Note*: Cronbach's alpha presented in the parentheses on the diagonal. Correlations represent raw correlations of manifest variables.

Abbreviations: CD = Career Decidedness, SE = Self‐Efficacy, t1 = time 1, t2 = time 2, t3 = time 3, t4 = time 4.

^a^ 1 = male and 0 = female.

^b^ 1 = positive and 0 = negative.

^c^ *p* < 0.05.

^d^ *p* < 0.01.

To explore developmental trends, we conducted repeated measurement ANOVAs on participants who completed all four waves (*n* = 89). Results indicated a significant linear increase in both self‐efficacy and career decidedness over time. As Mauchly's test indicated that the assumption of sphericity had been violated, χ2 (5) = 15.69, *p* = 0.008, degrees of freedom were corrected using Huynh‐Feldt estimates of sphericity (ε = 0.917). The overall effect of time on NEETs' self‐efficacy and career decidedness was significant at the 0.05 level, *F*(2.75, 242.18) = 18.13, *p* = < 0.001, partial η2 = 0.17. Post hoc analyses qualified these findings for self‐efficacy, identifying significant changes only from t2 to t3 (*p* < 0.001), yet not at the start (*p* = 0.235) and towards the end of the program (*p* = 0.124). In contrast, career decidedness showed consistent growth across all intervals (*p* = 0.007 for t1 to t2; *p* = 0.002 for t2 to t3, and *p* = 0.005 for t3 to t4). Following established guidance (Becker 2005; Becker et al. 2016), candidate background controls were screened and, given their negligible incremental value, excluded from the final models.

### 2.2 Hypothesis Testing

We tested our hypotheses in a sequence of nested structural equation models (see Table 2 for fit indices and comparisons). Model fit improved incrementally from Model A through Model D, but not when we added the random intercepts in Model E, suggesting Model D to be the model of choice (see Table 3 for the standardized regression weights).

**Table 2:** Model fits for study models.

Model	χ²			CFI	TLI	SRMR	RSMEA		TRd	
	Value	df	*p*				Value	90% CI	Δ χ² (Δ df)	*p*
Model A (correlational)	682.52	282	< 0.001	0.83	0.82	0.25	0.073	[0.066, 0.080]		
Model B (predictive)	676.83	279	< 0.001	0.83	0.82	0.25	0.073	[0.066, 0.081]	5.62 (3)	0.132
Model C (predictive)	669.26	276	< 0.001	0.83	0.82	0.22	0.073	[0.066, 0.081]	7.42 (3)	0.060
Model D (CLPM)	378.53	270	< 0.001	0.95	0.95	0.08	0.039	[0.029, 0.048]	378.79 (6)	< 0.001
Model E (RI‐CLPM)	398.32	267	< 0.001	0.94	0.94	0.07	0.043	[0.034, 0.052]	0.88 (3)	0.831

*Note*: Model A–C = measurement model of latent variables of Self‐Efficacy and Career Decidedness. TRd = Satorra‐Bentler Scaled Chi‐Square Difference.

**Table 3:** Standardized regression weights for cross‐lagged SEM model of self‐efficacy and career decidedness over time (Model D).

Path	Estimate	Standard Error	*p* value
Autoregressive Paths			
CD T1 → CD T2	0.59	0.09	< 0.001**
CD T2 → CD T3	0.44	0.13	0.001**
CD T3 → CD T4	0.74	0.12	< 0.001**
SE T1 → SE T2	0.79	0.06	< 0.001**
SE T2 → SE T3	0.75	0.10	< 0.001**
SE T3 → SE T4	0.61	0.13	< 0.001**
Cross‐lagged Paths			
CD T1 → SE T2	−0.10	0.07	0.161
SE T1 → CD T2	0.12	0.10	0.199
CD T2 → SE T3	−0.09	0.11	0.392
SE T2 → CD T3	0.01	0.13	0.962
CD T3 → SE T4	0.08	0.13	0.534
SE T3 → CD T4	−0.13	0.11	0.228
Predicting the outcome			
CD T4 → NEETs' exit	0.45	0.11	< 0.001**
SE T4 → NEETs' exit	0.00	0.10	0.967

Abbreviations: CD = career decidedness; SE = self‐efficacy.

* *p* < 0.05

** *p* < 0.01.

Hypothesis 1a *Cross‐Sectional Associations*. As expected, self‐efficacy and career decidedness showed positive correlations at all four measurement points. Except for slight variations at T4 (discarded Models C, E) and T3 (discarded Model E), this crosssectional relationship held across all models and hypotheses, including the best‐fitting Model D, thus supporting Hypothesis 1a.

Hypothesis 1b *Self‐Efficacy Predicting Later Career Decidedness*. However, when adding the paths from self‐efficacy at one time‐point to career decidedness at the following time‐point in Model B, self‐efficacy did not show the proposed predictive relation to subsequent career decidedness at any time interval (or in any of the models B to E including this relationship), thus rejecting Hypothesis 1b.

Hypothesis 1c *Career Decidedness Predicting Later Self‐Efficacy*. When adding the paths from career decidedness at one time‐point to self‐efficacy at the following time‐point in Model C, career decidedness only fostered subsequent self‐efficacy from third to fourth measurement point, but not during either of the earlier measurement periods. Also, the relationship between third and fourth measurement point did not hold in subsequent models, thus largely rejecting Hypothesis 1c.

Hypothesis 1d *Controlling for Autoregressive Stability (CLPM)*. When adding the autoregressive paths for both self‐efficacy and career decidedness in the CLP‐Model D, thus controlling for participants' starting values, no cross‐lagged relationships emerged either, yielding the same results as reported by Creed et al. (2006) and thus rejecting Hypothesis 1 d. Autoregressive paths, however, held stable across all measurement points.

Hypothesis 1e *Controlling for Between‐Person Variance (RI‐CLPM)*. When controlling for effects of between person variances in Model E by adding the random intercepts, we further found no cross‐lagged effects, thus rejecting Hypothesis 1e.

***Hypotheses 2a and 2b:** Prediction of exiting NEET Status*. Rejecting Hypothesis 2a, Self‐efficacy at the fourth measurement point did not show any relationship to NEETs' exit status in any of the models. Career decidedness at the fourth measurement point, in turn, positively related to NEETs' exit status across all five models, thus supporting Hypothesis 2b. Standardized regression weights for Hypothesis 2 are depicted in Table 4.

**Table 4:** Standardized regression weights for SEM models of self‐efficacy, career decidedness and NEETs’ exit from NEET status (Outcome).

Model	H2a, H2b			
	Path	Estimate	Standard Error	*p* value
Model A	SE T4 → Outcome	−0.02	0.10	0.829
	CD T4 → Outcome	0.49	0.11	< 0.001**
Model B	SE T4 → Outcome	−0.02	0.11	0.827
	CD T4 → Outcome	0.49	0.12	< 0.0011**
Model C	SE T4 → Outcome	−0.02	0.10	0.869
	CD T4 → Outcome	0.48	0.11	< 0.0011**
Model D	SE T4 → Outcome	0.00	0.10	0.967
	CD T4 → Outcome	0.45	0.11	< 0.0011**
Model E	SE T4 → Outcome	0.01	0.22	0.959
	CD T4 → Outcome	0.52	0.24	0.0341*

Abbreviations: CD = career decidedness; SE = self‐efficacy.

* *p* < 0.05

** *p* < 0.01.

Summarized, self‐efficacy and career decidedness covaried with one another within assessments but had no influence on each other across time. Participants' end‐of‐program career decidedness had a positive influence on exit from NEET status, with no such effect emerging for self‐efficacy.

## 3 Discussion

This study examined the dynamic interplay between 264 NEETs' career‐related self‐efficacy and career decidedness across time and the effects of both these pivotal constructs on NEETs successfully exiting the NEET status. While general (Ajzen 1991; Ajzen and Fishbein 1980) and career‐specific theorizing (e.g., Gottfredson 2005; Savickas 2005; Lent and Brown 2013) largely assume directional relationships between self‐efficacy and career decidedness, our findings challenge this assumption. We replicated consistent positive cross‐sectional associations between the two variables, but found no longitudinal (cross‐lagged) effects. Results further indicate that NEETs' final career decidedness positively predicted their exit from NEET status, whereas self‐efficacy showed no such effects.

These findings are noteworthy for two reasons: First, they contradict core assumptions of dominant career development theories (e.g., Gottfredson 2005; Savickas 2005; Lent and Brown 2013), which posit that self‐efficacy not only covaries with but actually facilitates career decisions. These findings are also relevant given the scarcity of suitable longitudinal studies on the question, which further yielded inconclusive results and allowed for alternative interpretations. Our study extends this literature and solidifies the questions surrounding the power of self‐efficacy in fostering career decidedness and NEETs' subsequent ability to leave the NEET status. Second, the study provides new insights into the developmental processes of one of the most vulnerable youth populations, NEETs, which has rarely been the focus of multi‐wave longitudinal research. Unlike most previous studies, which rely on more privileged student samples (Lent et al. 2019; Sheu 2023) and cross‐sectional designs (Betz and Voyten 1997; Lent et al. 2016; Taylor and Betz 1983), this study follows NEET youth over four time points during a transitional phase. This allows us to better understand how career‐related self‐efficacy and decidedness develop over time in a group that often faces great uncertainty and instability.

### 3.1 Cross‐Lagged Effects Between Self‐Efficacy and Career Decidedness

The current study calls into question the role of self‐efficacy as proposed in dominant career theories. Dominant theories such as the SCCT (Lent and Brown 2013; Lent et al. 1994; Lent et al. 1999) propose that self‐efficacy fosters the occupational decision‐making process, resulting in higher levels of career decidedness. Indeed, our cross‐sectional data (Model A) align with this assumption. Yet, this relationship did not hold longitudinally (Model B to E) and appears to be solely due to a combination of autoregressive and cross‐sectional relationships (as included in the CLP‐Model D), suggesting no stable temporal dynamics between self‐efficacy and career decidedness. While counter‐theoretical, this finding mirrors and extends results from Creed et al. (2006) and from Lent et al. (2019), wave two to three, who also found no cross‐lagged relationships between these variables. Creed et al. (2006) advised several changes in future study designs, such as shorter time lags, more frequent measurement points, and an older sample. Yet, despite following these recommendations, our data still produced the same null‐effect.

One possible explanation might indeed be methodological: It is conceivable that also our time‐lag, even though shorter than in previous studies, was not ideal (Dormann and Griffin 2015). Creed et al. (2006) had surveyed regular students in grades 8 and 10, thus with a long time‐lag and at a time when no imminent career decisions needed to be made. Lent et al. (2019) surveyed college students roughly 4 and 3 months apart, which might have contributed to their mixed findings. We surveyed young people during a transitional phase aimed at remedying a misgone school‐to‐work transition and chose a time‐lag of about 2.5 months. Yet, we, too, may have missed the optimal time‐lag to accurately capture shifts in self‐efficacy and career decidedness during this pivotal time for NEETs' career prospect. According to action regulation models like the Rubicon Model (Heckhausen and Gollwitzer 1987), people's focus shifts when transitioning from a pre‐decisional to a post‐decisional phase, from a wide motivational focus open to exploring different options to a narrow volitional focus. Before a decision, self‐efficacy may play a motivating role, supporting NEETs in forming intentions and committing to career‐related goals. After a decision, however, self‐efficacy may become more of a consequence of action execution and goal pursuit, rather than a primary predictor of further decisional outcomes. If therefore time intervals between measurements do not adequately align with the decision point or the surrounding phases, associations between self‐efficacy and career decidedness may appear weaker or more unstable.

Alternatively, and contrary to the commonly held assumptions, self‐efficacy may indeed not necessarily be a robust facilitator of career decidedness across time (Creed et al. 2006). Self‐efficacy, while not particularly low on average in our sample, may not be enough to support clear career decisions under the conditions faced by NEET youth. External barriers—such as limited access to education or training opportunities, structural constraints in the labor market, or a lack of social support—may restrict the degree to which young people can act on their beliefs. Also, it is conceivable that many NEETs lack the experience from the labor market to realistically estimate their dire standing in the labor market. In either case, this suggests that motivational beliefs alone may not be sufficient in contexts where opportunities are scarce or hard to access. Furthermore, following the descriptive variables, we saw indeed a change in value but a relative stability in rank‐ordering in self‐efficacy and career decidedness from each measurement point to the next. It is thus conceivable that both variables change—but do so covaryingly, without one particularly influencing the other, with their correlational relationships across time being fully explained by crosslagged covariance and each variable's respective stability.

## 4 Exiting NEET Status

### 4.1 Self‐Efficacy

Based on theorizing (Lent and Brown 2013; Moynihan et al. 2003), we also expected self‐efficacy to predict NEETs' exit from their NEET status. Yet, the data showed no relationship between self‐efficacy at program completion and NEETs' exit. While conceptually unexpected, this null‐finding is actually not the first of its kind. That is, research—and specifically research with longitudinal designs—has also shown that self‐efficacy can have null or even adverse effects on the envisioned outcomes (Vancouver et al. 2002; Vancouver and Kendall 2006; Vancouver et al. 2014). For example, when people hold too high convictions of their own capabilities, they may reduce their goal‐related efforts, as they are convinced to reach the goal either way—and may thus ultimately fail to reach it. In the current study, the NEETs' average self‐efficacy was quite high to begin with, and possibly higher than warranted, given NEETs' objectively precarious position in the labor market. Thus, they may have put less effort into the search for an internship, an apprenticeship, a job or a place for further education than necessary out of a misguided belief that they will leave a good impression on a potential employer in any case. How this, in turn, influenced their actual performance in a job interview for securing employment warrants future research.

### 4.2 Career Decidedness

In line with the notion that a clear conviction of their aspirations for the future should help NEETs arrive at a positive career outcome, NEETs' career decidedness at the fourth measurement point indeed predicted their exit from the NEET status. This aligns with reasoning theories like the TRA (Ajzen and Fishbein 1980) and the TPB (Ajzen 1991) that emphasize the role of intentionality in shaping behavior. In the context of career decision‐making, career decidedness reflects a formed intention to pursue a specific vocational path. It also aligns with career construction theory (Savickas 2005), where career decidedness is seen as a support to vocational identity development, and with the self‐regulatory process outlined in the SCCT (Lent et al. 1994). The finding also aligns with prior work showing that career decidedness predicts work‐related outcomes such as organizational commitment (Earl and Bright 2007) and job search quality (Koen et al. 2010). In the NEET context, this is particularly important: knowing what one wants appears more consequential than believing in one's general capability to succeed.

## 5 Limitations and Directions for Future Research

Although we improved upon earlier designs, the 2.5‐month intervals between waves may still have missed critical psychological transitions. Future research could examine changes within self‐efficacy and career decidedness during the school to work transition with even shorter intervals, potentially even following young people on a daily basis with a diary study design or a controlled experimental design to possibly leverage causal effects and thus adequately capture the transitional phases of goal‐directed action (Heckhausen and Gollwitzer 1987).

Also, the quality of young peoples' motivation to search for employment or educational options might play a role, especially with the aforementioned potential adverse effects of high self‐efficacy. Motivation matters during job‐search (Da Motta Veiga and Gabriel 2016; van den Hee et al. 2020), and especially autonomy and autonomous motivation seem vital (Koen et al. 2016). However, participation in the interim program from which we recruited participants was mandatory, and not all participants were innately motivated to engage in career preparation. Possibly, self‐efficacy may only feed into career decisions under conditions of self‐direction and autonomy. Therefore, future research could explore the role of autonomous versus more controlled motivation in this process.

Relatedly, common theories also suggest that self‐efficacy not only directly facilitates the career decision process, but also indirectly by strengthening the expected outcomes associated with such a decision (e.g., Lent and Brown 2013). While not proposed in any of the theories outlined above, it is conceivable that self‐efficacy may not always predict career decidedness, but only, if the decisional options are attractive. If a NEET, however, faces a highly stratified system as in Germany and expects available jobs to be undesirable (e.g., physically strenuous with undesirable working hours and possibly underpaid), they may not be motivated to pursue any of these options, no matter their self‐efficacy to meet those options' requirements.

This also highlights the role of context. NEETs represent a very diverse group among the unemployed (Carcillo et al. 2015) and different countries and economic circumstances may elicit different demands and opportunities for this population. The German system for vocational education and training is particularly stratified (Berloffa et al. 2018; Mascherini 2018; Protsch and Solga 2016; OECD 2025); future research should thus examine how NEETs with limited schooling can access flexible, less‐credential‐dependent work settings that provide meaningful experience to offset educational gaps, while also probing how self‐efficacy and career decidedness operate under these conditions.

## 6 Practical Implications

Our results suggest that career decidedness, more than self‐efficacy, plays a key role in facilitating NEETs' exit from NEET status and thus their reintegration into education or employment. Therefore, while many interventions target motivational beliefs, our findings suggest that helping youth form clear and realistic career goals may be more effective—particularly in contexts where opportunities are limited or access is constrained. Policymakers and educators should consider strengthening the role of career guidance and exploration, ideally before students exit the school system, and should help youth identify concrete paths, rather than focusing solely on building confidence. Given the self‐perpetuating nature of being NEET, interventions could particularly focus on youths from educationally less fortunate backgrounds to gain more experience and thus help them access better options. Our findings underscore the need for interventions, focused on career development, to take contextual constraints, such as being NEET, more seriously and point toward the importance of enriching motivational support with structural approaches that improve access to career opportunities.

Finally, the setting examined here, despite its compulsory nature, showed increases in both, NEETs self‐efficacy and career decidedness from T1 to T4. As career decidedness proved valuable for exiting NEET status, the setting studied here represents a viable approach for bettering young peoples' outlook on the future by helping them clarify their goals and navigate structural constraints—offering more than just motivation, but direction. This thought not only pertains to NEETS, as data from the OECD (2025) show that nearly half of 15‐year‐old students in Germany report high levels of career uncertainty, and almost 60% feel insufficiently prepared by school to take up a job. This widespread lack of clarity about future trajectories may limit young people's ability to explore and engage with available opportunities—an issue that closely aligns with the findings of the present study. Based on our study's results, future interventions could focus more on their practical, hands‐on methods instead of trying to foster more motivation—or figuratively speaking, it might be more beneficial for young people to know which harbor they want to reach than believing that they can sail to just about anywhere.

## 7 Conclusion

This longitudinal study followed 264 young people not in employment, education, or training over the course of 10 months to see to whether they found a port to sail towards. Self‐efficacy, contrary to our expectations, covaried with but showed no causal influence on career decidedness or NEETs' exit from NEET status. Only career decidedness predicted exit from NEET status. These findings suggest that, particularly in low‐opportunity contexts, clarity about one's career path may matter more than confidence in one's abilitites. Strengthening career decidedness may thus be a key lever in supporting vulnerable youth through the school‐to‐work transition.

## Ethics Statement

Ethics approval was obtained by the participating university's Research Ethics Board.

## Conflicts of Interest

The authors declare no conflicts of interest.

## Supporting information

Supplement A_Setting Sails for Your Harbor.

## Data Availability

Data used in the manuscript is available upon request.

## Endnotes

1 We thank Reviewer two for pointing our attention to the fitting conceptualization of reasoning approaches suitable for this paper.

2 We carefully surveyed databases such as PsychNet, Google Scholar, EBSCOhost, JUSTfind, and Web of Science. Selection criteria were a longitudinal design with the inclusion of two or more measurement points and either a cross‐lagged design or baseline control. As for variables we only searched for studies including measures of either career decidedness or career indecision in combination with self‐efficacy. While quite a few studies included one or even several of our inclusion criteria, the only studies we could find that met all our criteria are reported in the following and represent the studies to which we have oriented ourselves most closely.

3 Large portions of the educational and vocational market particularly for young people are very structured in Germany. The most popular pathways of the STWT in Germany are the pursuit of further education in the form of a high‐school‐diploma or further university study, and the pursuit of part‐time vocational schooling besides on‐the‐job training, leading to the attainment of a vocationally qualified degree. When failing to secure a placement in time, the next respective opportunity usually only emerges with the following cohort a year later.
