# Supplementary material for: Setting Sails for Your Harbor: Navigating Beyond NEET Status Through Self‐Efficacy and Career Decidedness
Source: J Adolesc. 2025 Sep 16;98(1):200–12. doi: 10.1002/jad.70051 (PMC12780653; doi:10.1002/jad.70051)
Supplement: Supplementary file 1 — Supplement A_Setting Sails for Your Harbor. [file JAD-98-200-s001.docx]

# Supplemental

**Computation of the RI-CLPM**

The RI-CLPM (Hamaker, 2018; Hamaker et al., 2015) represents an expansion of the conventional cross-lagged panel model (CLPM), designed to differentiate between enduring, trait-like parts among units (in our instance: persons), and the variability of within-unit-changes, ensuring that lagged relationships exclusively relate to the latter. This method answers to the call for accounting not only for stability, but also for ensuring that stability across measures that is due to trait-like effects is not misinterpreted as effects of temporal causality within persons/units (Hamaker et al., 2015). Like the CLPM, the RI-CLPM includes latent variables for each measurement occasion, as well as autoregressive paths. Yet, the CLPM accounts for temporal stability via autoregressive paths, thus implying that all persons/units vary around the same mean, thus failing to model between-unit differences in trait-level which might influence individuals. The RI-CLPM, in contrast, accounts for such differences using *random intercepts* (Hamaker et al., 2015). Random intercepts represent stable individual differences in the measured constructs, which are not influenced by time or intervention, thus separating a between- from a within-person level. The cross-lagged relationships within the RI-CLPM hence depict reciprocal influences over time on a within-person level.

For a detailed description of how to compute a RI-CLPM, please refer to Mulder and Hamaker (2021). In order to compute a RI-CLPM for multiple indicators, as we did with our data, one first has to establish measurement invariance over time (Hamaker, 2018) for meaningfully comparing the latent variables over measurement points. To ensure this, we first computed a measurement model, establishing four latent factors for each self-efficacy beliefs and career decidedness, to depict the four time-points. Each latent variable was estimated using three parcels comprised of the observed variables. We then used each of the four latent factors for each self-efficacy beliefs and career decidedness from the measurement model to reflect the between person stability, thus creating two latent random intercepts; one for self-efficacy beliefs and one for career decidedness.

To test the prerequisite of measurement invariance we constrained the factor loadings of the measurement model to be invariant over time, testing for weak factorial invariance. This model is nested under the regular measurement model, where no constraints are imposed. Thus, to test whether weak factorial invariance holds within the model, chi-square difference testing will suffice. We tested the difference using the Satorra-Bentler Scaled Chi-Square (Satorra & Bentler, 2010), supporting the assumption of weak factorial invariance. This means that when comparing the constrained model with the one where factor loadings were allowed to freely correlate, factor loadings are not significantly different from each other (Hamaker, 2018).

We further tested for strong factorial invariance by constraining the intercepts, i.e. the means, identifying the latent factors of the measurement model, to be invariant over time. Further chi-square difference testing revealed a significant difference in chi-square, meaning that the data did not hold to strong factorial invariance (Hamaker, 2018). By allowing the intercepts to freely vary over time, this means that there seems to be change at population level which varies over the course of the measurement points (Mulder & Hamaker, 2021). This actually aligns with our finding of increasing self-efficacy and career-decidedness across the course of the educational program (see Table 1 and Figure 1 in the manuscript).

Regarding the RI-CLPM tested in the current study (Model E), results of weak but not of strong factorial invariance led to us being able to constrain the factor loadings for the latent factors as being invariant over time, but not being able to constrain the intercepts. Using the measurement model and the thus established weak factorial invariance, we specified the rest of the RI-CLPM. Table 1 shows the model comparisons for the invariance testing of the RI-CLPM.

**Statistical Performance of the RI-CLPM**

While the justification to compute a RI-CLPM seems evident from a statistical standpoint to clearly separate between- from within-person variance and to gain deeper insights on a content-level into the relationship between self-efficacy and career decidedness, we also saw that the RI-CLPM brought no additional value in terms of explanation of variance over the regular CLPM. This raises the question whether it would be necessary to separate between- and within-person variance.

In our instance, the RI-CLPM (Model E) brought no added value in terms of fit over the classic CLPM (Model D), and thus statistical rigor advises to go with the simpler (CLPM) model instead. As the use of either model did not alter the interpretation of our results regarding Hypotheses 1 and 2 (i.e., we still found cross-sectional but no cross-lagged effects between self-efficacy and career decidedness with career decidedness at T4 predicting NEETs status upon program completion), the choice of model further bears no consequence for our conclusions.

Yet, we would still like to note that in Model E, we lost autoregressive stability when controlling for the between-person factor. This is something one needs to be aware of when using this statistical procedure. Between-person effects can be attributed to changes within, thus (over)estimating effects of change where variables are stable. That said, such loss of autoregressive effects from the CLPM to the RI-CLPM, with the latter being nested under the former, is indeed no uncommon phenomenon (Mulder & Hamaker, 2021). While the traditional CLPM captures within- and between-person effects within the autoregressive parameters, the RI-CLPM distinguishes between both, using the random intercepts. Thus, autoregressive parameters in the RI-CLPM no longer represent stable, “re-test”-like constructs “but […] account for additional moment-to-moment stability (i.e., inertia or carry-over) of the within-unit fluctuations over time” (Mulder & Hamaker, 2021, p. 642). Traditionally, the relationship of the cross-lagged parameters between the traditional CLPM and the RI-CLPM can differ (Mulder & Hamaker, 2021), accounting for more than mere change in the variables. Within our paper, this could speak for stronger effects of between-person differences concerning self-efficacy and career decidedness and smaller effects concerning the within-person fluctuation over the four measurement points, which could also explain the loss of the autoregressive stability, when the moment-to-moment-stability we saw in the CLPM is indeed because of between-person stability.

References

Hamaker, E. L. (2018). *How to run a multiple indicator RI-CLPM in Mplus*. https://www.researchgate.net/publication/328095575_How_to_run_a_multiple_indicator_RI-CLPM_in_Mplus

Hamaker, E. L., Kuiper, R. M., & Grasman, R. P. P. P. (2015). A critique of the cross-lagged panel model. *Psychological Methods*, *20*(1), 102–116. https://doi.org/10.1037/a0038889

Mulder, J. D., & Hamaker, E. L. (2021). Three Extensions of the Random Intercept Cross-Lagged Panel Model. *Structural Equation Modeling: A Multidisciplinary Journal*, *28*(4), 638–648. https://doi.org/10.1080/10705511.2020.1784738

Satorra, A., & Bentler, P. M. (2010). Ensuring Positiveness of the Scaled Difference Chi-square Test Statistic. *Psychometrika*, *75*(2), 243–248. https://doi.org/10.1007/s11336-009-9135-y

**Table 1**

*Model fits and model comparisons for invariance testing.*

| Model | χ² | | | CFI | TLI | SRMR | RSMEA | | TRd | *p*-value for TRd |
| --- | --- | --- | --- | --- | --- | --- | --- | --- | --- | --- |
|  | Value | df | *p* |  |  |  | Value | 90% CI |  |  |
| Model A (configural invariance) | 799.23 | 252 | <.001 | 0.76 | 0.74 | .28 | .091 | [0.084, 0.099] | — | — |
| Model B (weak factorial invariance) | 814.99 | 264 | <.001 | 0.76 | 0.75 | .29 | .090 | [0.083, 0.097] | 16.63 | .164 |
| Model C (strong factorial invariance) | 906.66 | 276 | <.001 | 0.73 | 0.73 | .29 | .094 | [0.087, 0.101] | 94.10 | <.001 |
| Model D (RI-CLPM) | 447.11 | 311 | <.001 | 0.95 | 0.94 | .08 | .041 | [0.032, 0.049] | — | — |

*Note.* Model A – C = measurement model of latent variables of Self-Efficacy and Career Decidedness. TRd = Satorra-Bentler Scaled Chi-Square Difference. Model D is based on Model B with addition of Random Intercepts, Internships and Employment Outcome.
